# A positive feedback loop between EZH2 and NOX4 regulates nucleus pulposus cell senescence in age-related intervertebral disc degeneration

**DOI:** 10.1186/s13008-020-0060-x

**Published:** 2020-02-01

**Authors:** Chang Liu, Libangxi Liu, Minghui Yang, Bin Li, Jiarong Yi, Xuezheng Ai, Yang Zhang, Bo Huang, Changqing Li, Chencheng Feng, Yue Zhou

**Affiliations:** Department of Orthopedics, Xinqiao Hospital, Army Medical University, Xinqiao Main Street 183, Shapingba District, Chongqing, People’s Republic of China

**Keywords:** Intervertebral disc degeneration, Nucleus pulposus cell senescence, Epigenetic histone modification, Wnt/β-catenin signaling pathway

## Abstract

**Background:**

The senescence of nucleus pulposus (NP) cells plays a vital role in the pathogenesis of intervertebral disc (IVD) degeneration (IDD). NADPH oxidase 4 (NOX4)-associated oxidative stress has been shown to induce premature NP cell senescence. Enhancer of zeste homolog 2 (EZH2) is a crucial gene regulating cell senescence. The aim of this study was to investigate the roles of EZH2 in NOX4-induced NP cell senescence and a feedback loop between EZH2 and NOX4.

**Results:**

The down-regulation of EZH2 and the up-regulation of NOX4 and p16 were observed in the degenerative discs of aging rats. EZH2 regulated NP cell senescence via the H3K27me3-p16 pathway. Also, EZH2 regulated the expression of NOX4 in NP cells through the histone H3 lysine 27 trimethylation (H3K27me3) in the promoter of NOX4 gene. Furthermore, NOX4 down-regulated EZH2 expression in NP cells via the canonical Wnt/β-catenin pathway.

**Conclusions:**

A positive feedback loop between EZH2 and NOX4 is involved in regulating NP cell senescence, which provides a novel insight into the mechanism of IDD and a potential therapeutic target for IDD.

## Background

Low back pain is one of the most common reasons why people ask for sick leave, thus placing an enormous burden on the social healthcare and insurance systems [1]. Low back pain has become a major cause of disability [2] and affects 80% of the population; importantly, low back pain is closely related to the severity of the degeneration of intervertebral discs (IVDs) [3, 4]. The persistent load on the spine due to upright posture seems to make people more susceptible to intervertebral disc degeneration (IDD) than other animals [5]. A pilot study has shown that nearly ninety percent of individuals over the age of 50 suffer IDD, which suggests IDD progresses with age [6].

Nucleus pulposus (NP) cell senescence plays a crucial role in the pathogenesis of IDD. The senescence-associated secretory phenotype (SASP) of NP cells includes a large number of cytokines, chemokines and matrix proteases. These factors not only promote extracellular matrix (ECM) degradation but also induce the death or senescence of adjacent NP cells [7]. Consequently, senescent NP cells disturb the homeostasis of IVDs to induce IDD [8].

NADPH oxidase 4 (NOX4) responds to various stresses to regulate ROS production and oxidative stress in various cells [9–11]. Oxidative stress induced by NADPH oxidase 4 (NOX4) is a major cause of NP cell senescence [12]. NOX4 generates ROS to cause DNA damage and subsequently activates MAPK and NF-κB signaling to induce NP cell senescence via the p53-p21-Rb and p16-Rb pathways [12]. This result suggested that NOX4 participates in the occurrence and development of IDD. However, further insights into the mechanism by which NOX4 promotes NP cell senescence have not been fully elucidated thus far.

Enhancer of zeste homolog 2 (EZH2) is a primary active component of polycomb repressive complex 2 (PRC2), which suppresses gene expression via compressing chromatin. EZH2 is a lysine methyltransferase and results in histone H3 lysine 27 trimethylation (H3K27me3) to suppress gene expression [13, 14]. Recent studies have reported that EZH2 downregulation causes PRC2 separation from the DNA replication fork in the early stage and consequently activates the DDR-ATM-p53-p21 pathway to promote cell senescence. At the later stage of EZH2 downregulation, H3K27me3 is demethylated, which upregulates p16 and SASP-related genes [15–17]. Moreover, EZH2 has been shown to be associated with intracellular oxidative stress [18, 19]. These results suggest that EZH2 plays potential roles in cell senescence induced by oxidative stress.

The aim of this study was to determine the roles of EZH2 in NP cell senescence. In addition, the interaction between EZH2 and NOX4 and the mechanism underlying this interaction were investigated herein. In light of histone modifications by EZH2, we focused our experiments on the epigenetic mechanism through which EZH2 regulates NOX4 expression in NP cells. In addition, we sought to examine whether NOX4 regulates EZH2 expression in NP cells. We hypothesized that a feedback loop between EZH2 and NOX4 regulates NP cell senescence and affects the IDD process. The results of the current study provide further understanding of the pathogenesis of NP cell senescence and IDD.

## Results

### The downregulation of EZH2 and upregulation of NOX4 in degenerative discs from aging rats

**Fig. 1:**
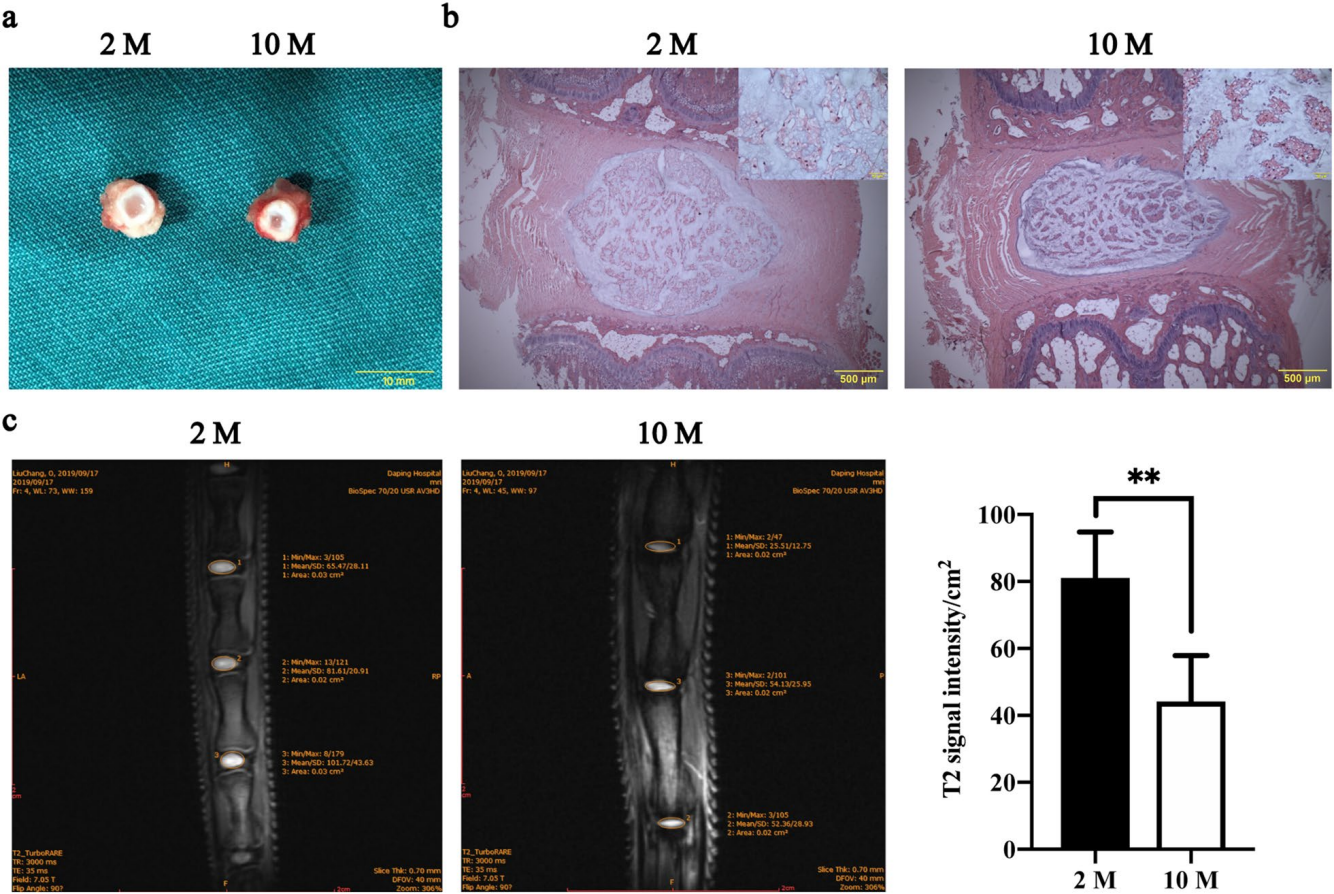
Caudal intervertebral discs (IVDs) of two-month-old (2 M) rats and ten-month-old (10 M) rats. **a** Cross section of caudal IVDs obtained from 2 M (left) and 10 M (right) rats. **b** H&E staining of caudal IVDs at different magnifications. **c** Micromagnetic resonance imaging (Micro-MRI) of caudal IVDs from 2 M and 10 M rats and T2 signal intensity of the nucleus pulposus (NP). The data are represented as the mean ± SEM (n = 9). **p < 0.01. H&E staining: hematoxylin and eosin staining

**Fig. 2:**
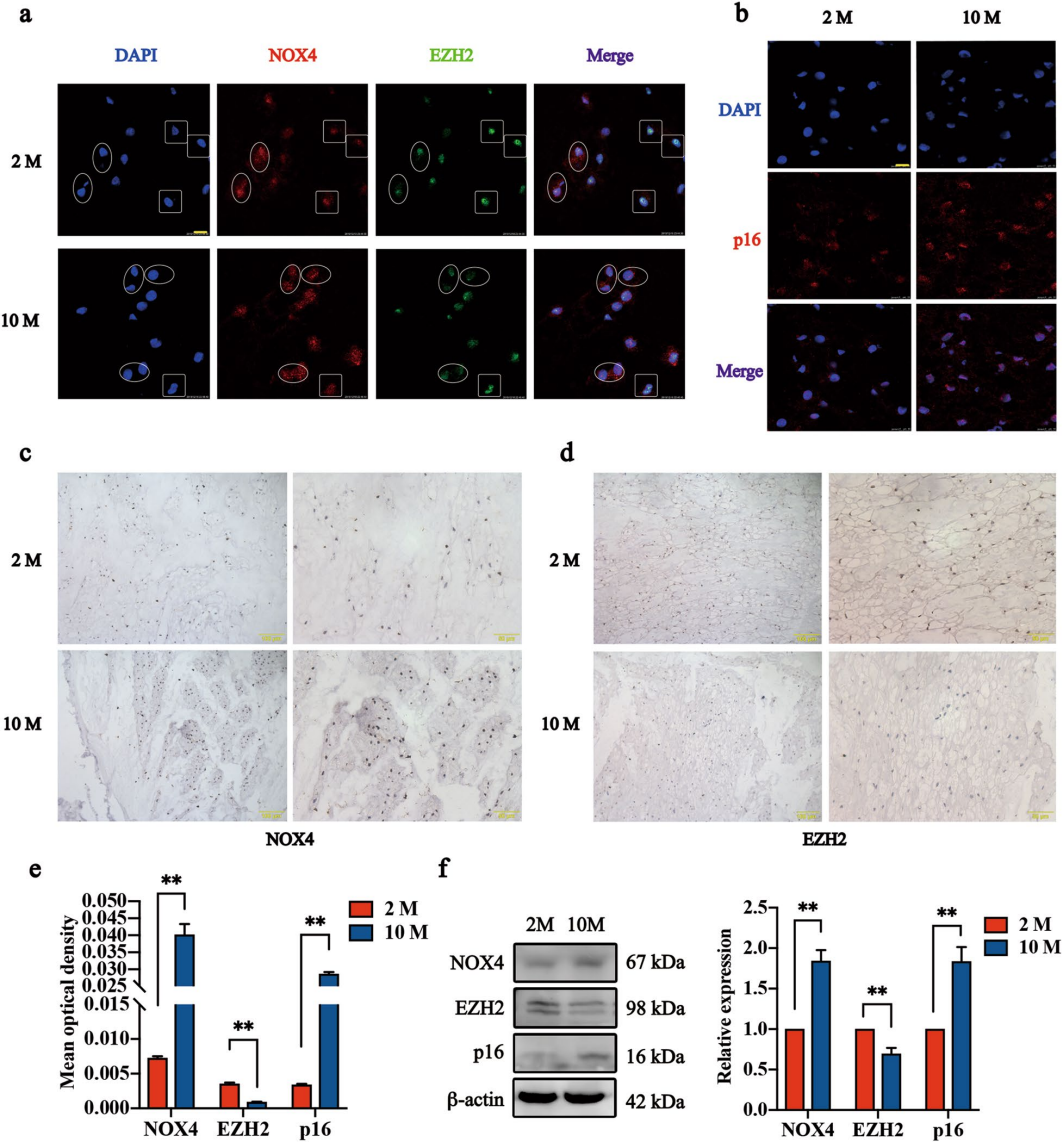
Inverse regulation of EZH2 and NOX4 with aging. Immunofluorescence images of frozen sections from NP tissues. Nuclei staining (DAPI), NOX4 and EZH2 staining (**a**) and p16 (**b**) staining are depicted in blue, green and red (n = 3). Cells in the ellipses were highly expressed NOX4 but lowly expressed EZH2. Cells in the squares were in the high expression of EZH2 and low expression of NOX4. Scale bar, 10 μm. NP paraffin sections stained with NOX4 (**c**), EZH2 (**d**), and p16 (Additional file 1: Figure S1) showed significant differences in 2 M and 10 M rats. **e** Show the mean OD of NOX4, EZH2, and p16 in NP sections. The data are represented as the mean ± SEM (n = 11). **f** Immunoblot analysis and OD analysis of NOX4, EZH2, and p16 in NP tissues extracted from 2 M rats and 10 M rats. β-actin was used as a loading control. The data are represented as the mean ± SEM (n = 4). **p < 0.01. *p < 0.05

**Fig. 3:**
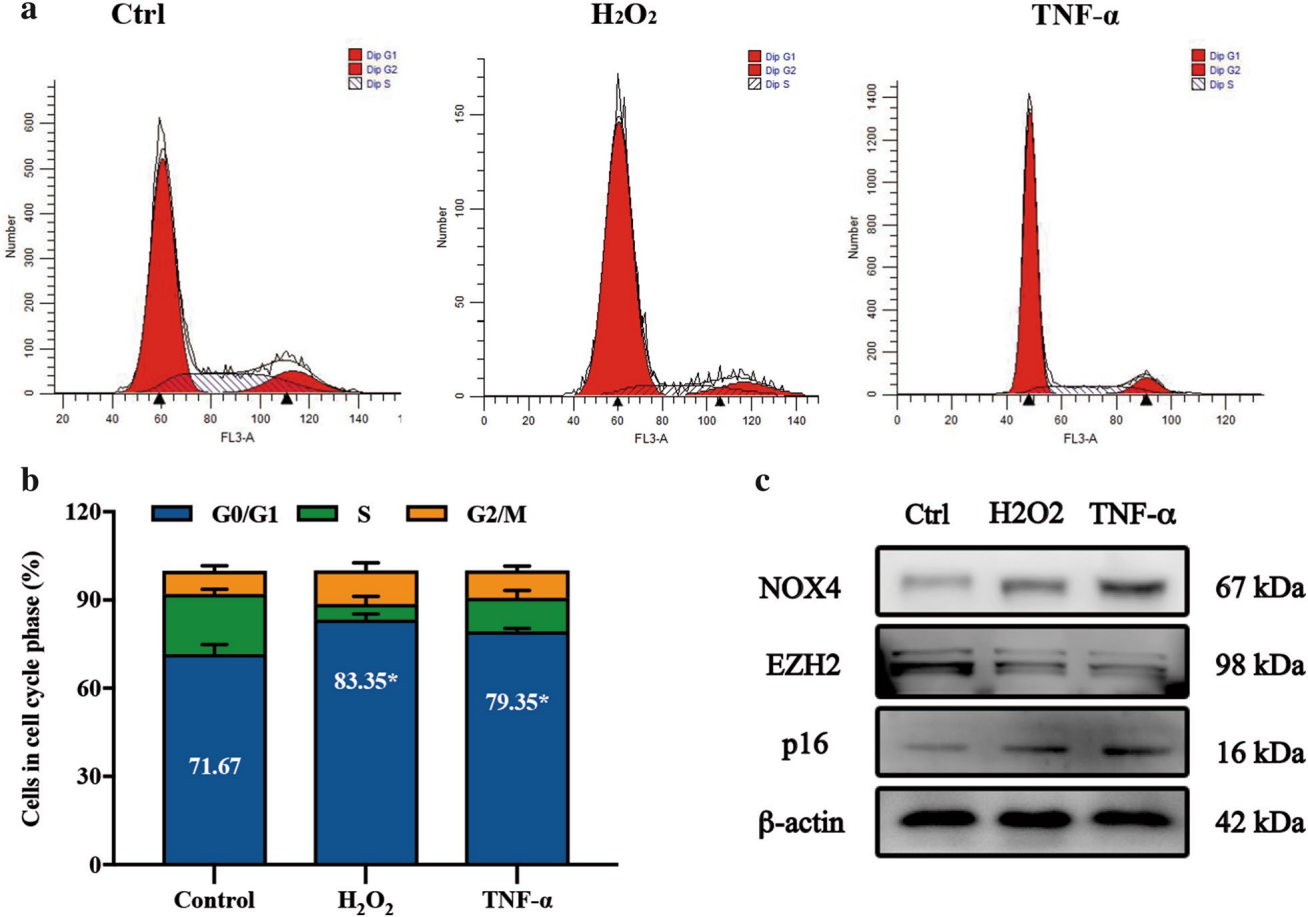
The expression of EZH2 and NOX4 in cell aging models induced by H_2_O_2_ and TNF-α. **a**, **b** The cells cycle distribution was determined by flow cytometry analysis. **c** Immunoblot analysis of EZH2, NOX4 and p16 in aging cells induced by H_2_O_2_ (100μM, 2 h) and TNF-α (30 ng/ml, 72 h). The data are represented as the mean ± SEM (n = 3). *p < 0.05

In general, NP tissue in 10 M rats became less hydrated and fibrotic compared to that in 2 M rats (Fig. 1a). According to H&E staining, the reduction or even disappearance of vesicles in NP cells was observed in 10 M rats. Additionally, the nuclei of the NP cells in 10 M rats became larger and rounder than those in 2 M rats (Fig. 1b). In addition, micro-MRI scanning showed that the T2 signal intensity of NP decreased significantly with rat aging (Fig. 1c), suggesting the occurrence of age-related IDD in rats. Moreover, significantly increased expression of NOX4 and p16 and significantly decreased expression of EZH2 were observed in the NP of degenerative discs using immunofluorescence (Fig. 2a, b, Additional file 1: Figure S1), immunohistochemistry (Fig. 2c–e, Additional file 2: Figure S2) and immunoblot analysis (Fig. 2f). Cells in high expression of NOX4 were companied by low expression of EZH2 (Fig. 2a, cells in ellipses). Cells in squares were opposite (Fig. 2a). And the mean fluorescence intensity of NOX4 in 2 M were lower than 10 M significantly, but EZH2 was opposite (Additional file 1: Figure S1). p16 was significantly upregulated in 10 M compared to 2 M (Fig. 2b). These findings were consistent with immunoblot analysis and immunohistochemistry. These results suggested the feedback loop between EZH2 and NOX4 existed and was involved in NP cell senescence. Not only in natural aging process, but these changes were also observed in cell aging models induced by H_2_O_2_ (100 μM, 2 h) and TNF-α (30 ng/ml, 72 h) (Fig. 3a–c). Previous studies have shown that NOX4 induced senescence in NP cells under oxidative stress through the MAPK and NF-κB pathways [12]. Therefore, the upregulation of NOX4 and p16 indicated more senescent NP cells in age-related degenerative discs. The significantly downregulated expression of EZH2 suggested a potential association between EZH2 and NP cell senescence, as well as IDD.

### Downregulation and inhibition of EZH2 lead to senescence in NP cells

EZH2 transcript levels were decreased in a replicative senescent model of human diploid fibroblasts [20]. Moreover, lower protein expression of EZH2 was confirmed in cells approaching replicative senescence [16].

**Fig. 4:**
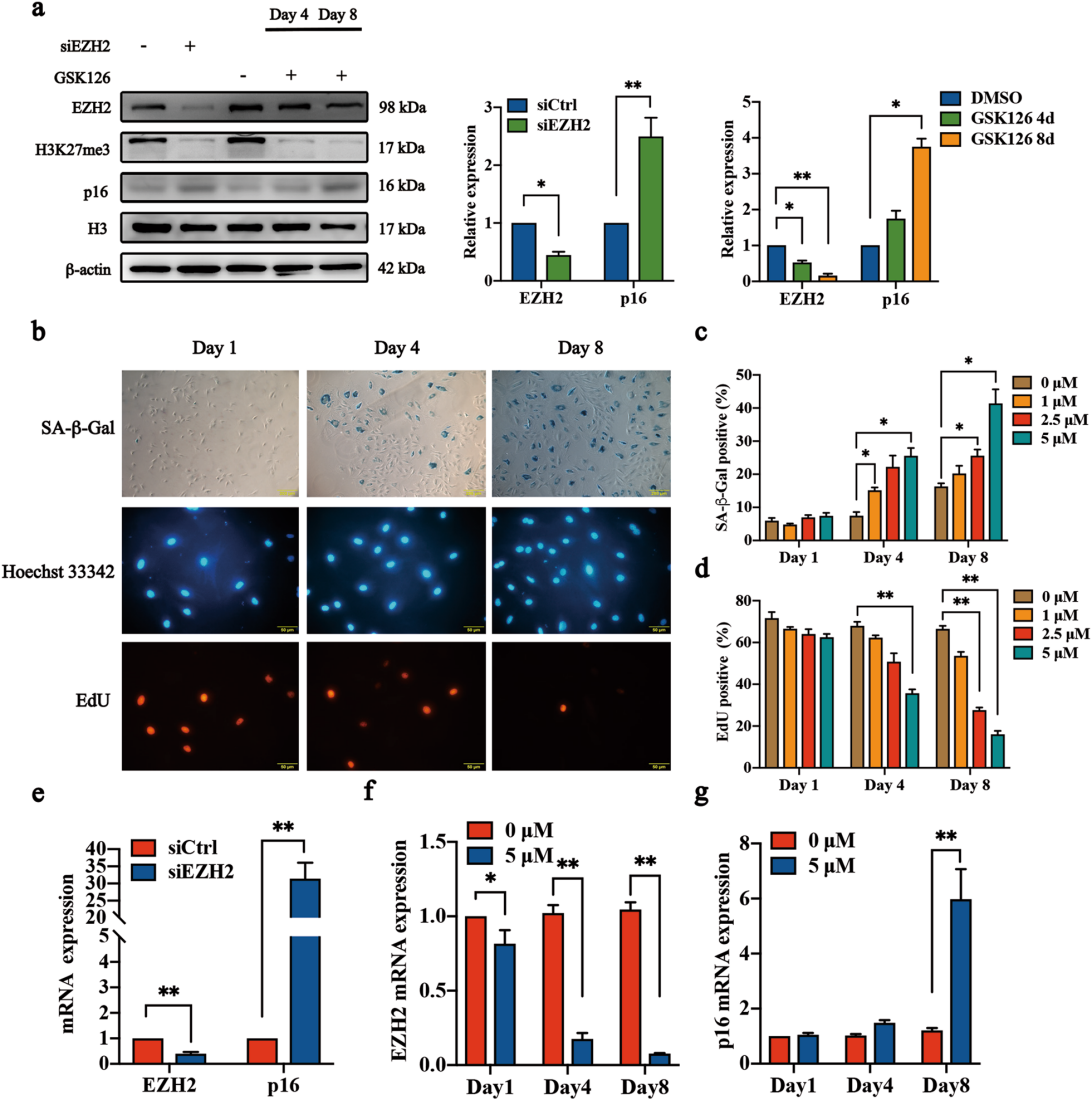
Downregulation of EZH2 and inhibition of EZH2 activity elicit NP cell senescence. NP cells were treated with GSK126 (0, 1, 2.5, or 5 μM) for 1 day, 4 days and 8 days. **a** Immunoblot analysis and OD analysis of EZH2, p16, and H3K27me3 in NP cells with EZH2 interference (siEZH2) or GSK126 treatment (5 μM for 8 days). β-actin or H3 was used as a loading control. The data are represented as the mean ± SEM (n = 3). **b** Images (cells treated with 5 μM for 1, 4, or 8 days) were captured under a phase-contrast microscope for SA-β-Gal and under a fluorescence microscope for EdU. **c**, **d** The percentages of Gal-positive cells and EdU-positive cells were calculated over a total of 800 cells treated with GSK126 (0, 1, 2.5, 5 μM) at different times. The data are represented as the mean ± SEM (n = 3). **e**–**g** RT-qPCR analysis of EZH2 and p16 in NP cells with EZH2 interference (siEZH2) or GSK126 treatment (5 μM for 8 days). The data are represented as the mean ± SEM (n = 3). NP cells transfected with scrambled siRNA control (siCtrl) were used as a control for siEZH2. DMSO was used as a control for GSK126. *p < 0.05, **p < 0.01

Herein, GSK126, a highly selective EZH2 methyltransferase inhibitor, inhibited H3K27 methylation and induced p16 expression in a time-dependent manner (Fig. 4a, g). Interestingly, we observed down-regulation of EZH2 induced by GSK126 (Fig. 4a, f). On the other hand, we transfected a lentivirus to interfere with the expression of EZH2 (siEZH2) in NP cells isolated from 2 M rats, and decreased expression of EZH2 was confirmed (Fig. 4a, e). More SA-β-Gal-positive cells and fewer EdU-positive cells were observed in NP cells treated with GSK126 and siEZH2 (Fig. 4b–d, Additional file 3: Figure S3). Meanwhile, siEZH2 caused a significant decrease in H3K27me3 expression and an increase in p16 expression 5 days after infection (Fig. 4a, e). Collectively, our research suggested that the downregulation of EZH2 and inhibition of methyltransferase activity induced premature senescence in NP cells.

### EZH2 regulated NOX4 expression in NP cells via H3K27me3

**Fig. 5:**
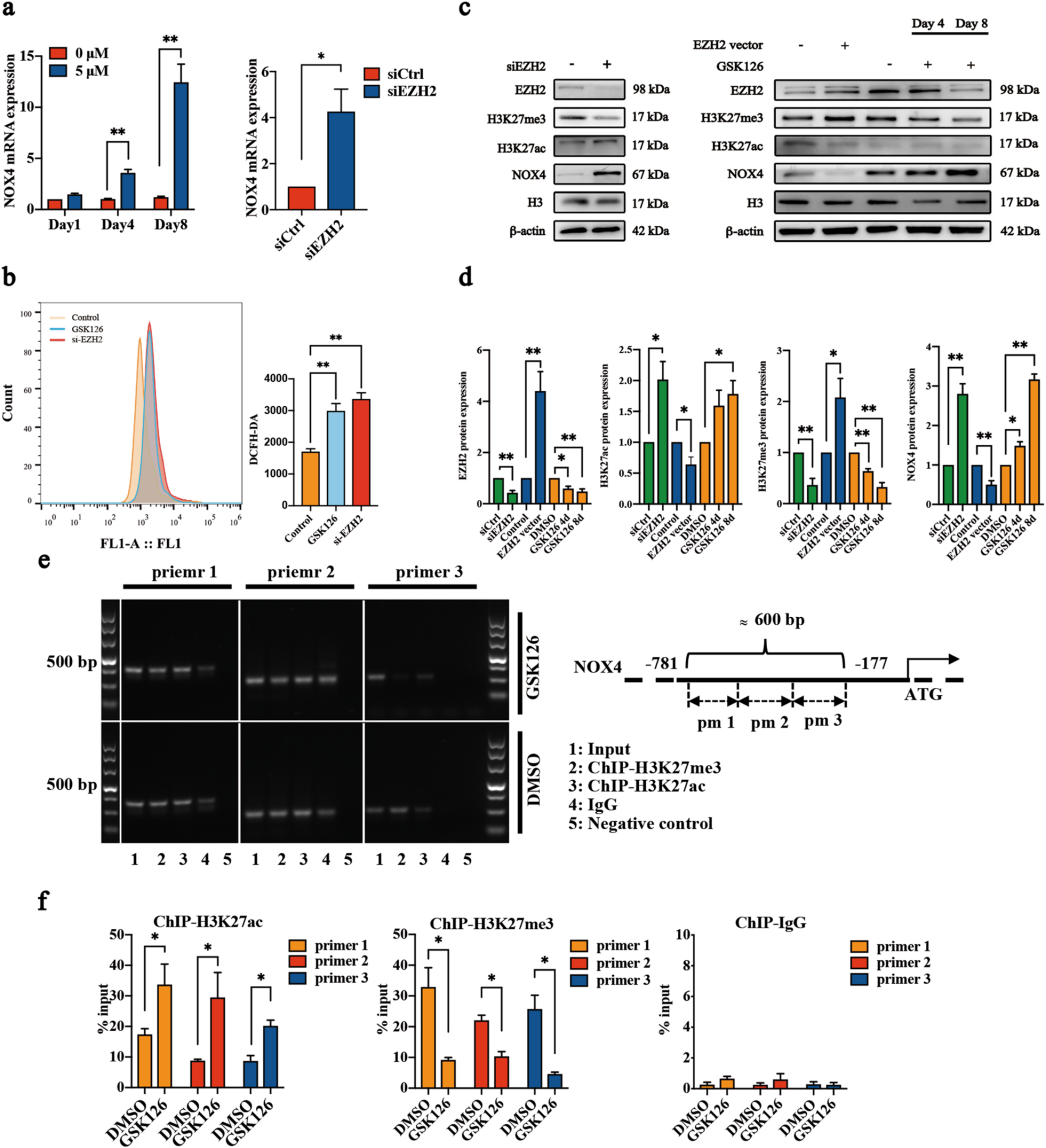
Regulation of NOX4 expression by EZH2. **a** RT-qPCR for NOX4 in NP cells with EZH2 interference (siEZH2) and GSK126 treatment (5 μM for 4 or 8 days). NP cells transfected with scrambled siRNA control (siCtrl) were used as a control for siEZH2. **b** Reactive oxygen species (ROS) levels measured using the DCFH-DA ROS-sensitive dye and flow cytometry. **c** Immunoblot analysis for EZH2, H3K27me3, H3K27ac, and NOX4 in NP cells overexpressing EZH2, treated with GSK126 or siEZH2 cells. β-actin and H3 were used as loading controls. NP cells transfected with empty lentivirus vectors were used as controls for groups overexpressing EZH2. DMSO was used as a control for the inhibitor. **d** OD analysis of the immunoblot analysis results in **c**. The data are represented as the mean ± SEM (n = 3). **e**, **f** ChIP-qPCR for H3K27me3 and H3K27ac at the proximal promoter regions of the NOX4 gene in cultured rat NP cells. Cells were treated with DMSO and GSK126 (5 μM) for 8 days, and primers were chosen for NOX4 constructive enhancer regions. Rabbit IgG was used as an IP control. The data are represented as the mean ± SEM (n = 3). *p < 0.05, **p < 0.01. *pm 1* primer 1, *pm 2* primer 2, *pm 3* primer 3

NOX4-dependent oxidative stress has been confirmed to participate in cell senescence induced by oncogenes and cytokines [21, 22]. Considering that EZH2 repressed gene expression by enhancing the posttranslational modification of histone 3, we further investigated whether EZH2 plays an important role in NOX4 transcription. Our results showed that siEZH2 or GSK126 significantly upregulated NOX4 expression in NP cells (Fig. 5a, c, d). And the ROS level was significantly increased (Fig. 5b). In contrast, the expression of NOX4 in NP cells was dramatically downregulated by EZH2 overexpression (Fig. 5c, d). Notably, the expression of H3K27me3 and H3K27ac in NP cells was also regulated by siEZH2, GSK126 and EZH2 overexpression (Fig. 5c, d). It was no coincidence that H3K27ac was up-regulated after knockdown of EZH2 [23]. Thus, we further mapped the H3K27me3 and H3K27ac marker around the NOX4 promoter by ChIP (Fig. 5e). The results confirmed the presence of the H3K27me3 marker around the NOX4 locus, and GSK126 significantly decreased the repressive H3K27me3 marker around the NOX4 locus in NP cells (Fig. 5e, f). Moreover, the regulation of EZH2 regulated the active H3K27ac marker in NP cells, and this marker was also mapped around the NOX4 locus. In addition, alteration of the active H3K27ac marker around the NOX4 locus was induced by GSK126 and si-EZH2 (Fig. 5c). The decrease of H3K27me3 and increase of H3K27ac were both involved in the upregulation of NOX4.

### NOX4 regulated the expression of EZH2 in NP cells through the canonical Wnt/β-catenin pathway

**Fig. 6:**
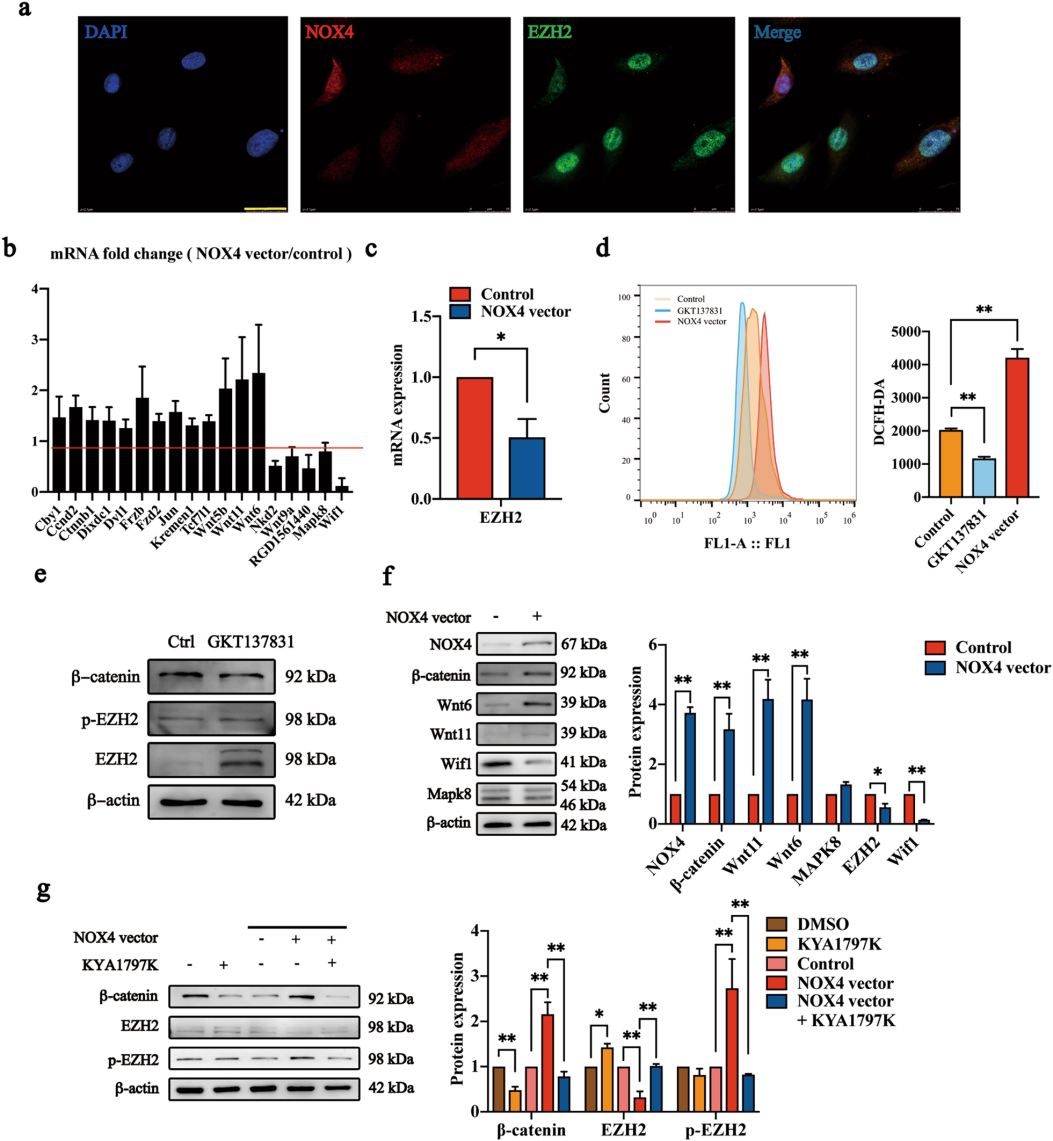
NOX4 regulates the expression of EZH2 and p-EZH2 through the canonical Wnt/β-catenin pathway. **a** NP cells were transfected with NOX4 vector for NOX4 overexpression and immunostained with antibodies for NOX4 (red) or EZH2 (green). The nuclei were stained with DAPI (blue). Scale bar, 25 μm. **b** The 18 genes which changed significantly in PCR array analysis (n = 3). The original PCR array analysis data are presented in Additional file 4: Table S1. **c** RT-qPCR analysis of EZH2 in NP cells overexpressing NOX4. The data are represented as the mean ± SEM (n = 3). **d** Reactive oxygen species (ROS) levels measured using the DCFH-DA ROS-sensitive dye and flow cytometry. **e** Immunoblot analysis for EZH2, p-EZH2 and β-catenin in cells treated with NOX4 inhibitor GKT137831 (20 μM, 24 h). **f** Immunoblot analysis for NOX4, β-catenin, Wnt6, Wnt11, Wif1, and Mapk8 in cells overexpressing NOX4. β-actin was used as a loading control. NP cells transfected with empty lentivirus vectors were used as a control. The data are represented as the mean ± SEM (n = 3). **g** Immunoblot analysis for NOX4, β-catenin, EZH2, and p-EZH2 in NP cells treated with the Wnt signaling pathway inhibitor KYA1797K (25 μM for 24 h), NOX4 vector, or both. β-actin was used as a loading control. DMSO was used as a control for the inhibitor. NP cells transfected with empty lentivirus vectors were used as controls for the groups overexpressing NOX4. The data are represented as the mean ± SEM (n = 3). *p < 0.05, **p < 0.01. *Wnt6* Wnt family member 6, *Wnt11* Wnt family member 11, *Wif1* Wnt inhibitory factor 1, *Mapk8* mitogen-activated protein kinase 8

EZH2 depletion was previously shown to promote oxidative stress-related cell death [18]. Based on these results, we hypothesized that EZH2 was regulated by NOX4 in a feedback manner. Herein, EZH2 in the nuclei of NP cells was found to be downregulated by NOX4 overexpression (Fig. 6a, c, g). Conversely, the phosphorylation of EZH2 was increased by NOX4 overexpression (Fig. 6g). Phosphorylation of EZH2 facilitated EZH2 degeneration and suppressed cell proliferation [24], and p-EZH2 has been confirmed to increase genotoxic stress-induced senescence [16]. Data on the further induction of cell senescence by excessive ROS after NOX4 overexpression have been previously published by our team [12], which was consistent with our results (Fig. 6d).

Studies have also shown that the expression of the Wnt/Myc pathway is inhibited after DNA damage, further reducing the transcription level of EZH2 [16]. Therefore, we hypothesized that the Wnt/β-catenin signaling pathway was involved in the regulation of EZH2 induced by NOX4. In fact, the expression of β-catenin in NP cells was increased by NOX4 overexpression (Fig. 6f), indicating the activation of the Wnt/β-catenin signaling pathway by NOX4. Besides, β-catenin was downregulated by NOX4 inhibition (GKT137831, 20 μM, 24 h) (Fig. 6e). And the expression of EZH2 was significantly increased. However, p-EZH2 showed no significant change after NOX4 inhibition (Fig. 6e). In the meanwhile, the level of ROS was significantly decreased (Fig. 6d). Furthermore, an RT^2^ profile PCR array for the rat Wnt signaling pathway was performed to determine the signaling molecules regulated by NOX4. The results showed that 13 genes were significantly upregulated, and 5 genes were downregulated (Fig. 6b). Furthermore, we immunoblotted for Wnt11, Wnt6, Wif1 and Mapk8, which showed the most noticeable change in PCR arrays. Most of these proteins were consisted with the PCR arrays, except Mapk8 (Fig. 6f). The original PCR array data are presented in Additional file 4: Table S1.

To investigate the roles of the Wnt signaling pathway in the decreased expression of EZH2 induced by NOX4 in NP cells, KYA1797K, a highly selective Wnt pathway inhibitor, was used. The expression of EZH2 and p-EZH2 in NP cells was not altered by KYA1797K (25 μM, 24 h). Nevertheless, the inhibitor KYA1797K suppressed the decreased EZH2 expression and increased p-EZH2 expression induced by NOX4 overexpression (Fig. 6g), suggesting that NOX4 regulates EZH2 and p-EZH2 expression via activating the canonical Wnt/β-catenin pathway, which forms a feedback loop.

## Discussion

Based on our micro-MRI results, the decrease of T2 signal intensity with aging indicated water loss. And cross section of caudal IVDs of 10 M rats showed more fibrotic changes compared to 2 M. To conclude, the age-related IDD does exist in rat caudal discs, thus establishing a reliable model for future studies. Besides, cells derived from the notch cord constitute the main component of NP cells [25], which also contribute importantly to maintaining disc function [26]. In our research, we observed vacuolated cells in paraffin sections. Nevertheless, the number of these vacuolated cells decreases over time, accompanied by significant morphological changes [26–29]. In the current study, we observed not only a decrease in vacuolated cells, but also appearance of larger nucleus with aging. And this process was companied by the down regulation of EZH2 and the upregulation of NOX4, p16. In fact, the high expression of EZH2 plays an important role in progenitor-state maintenance by silencing gene expression [30, 31]. In addition, EZH2 knockout induces cell senescence through early DNA damage and late epigenetic changes to p16 [16]. Therefore, we hypothesized that EZH2 loss leads cells to inactivity and then ultimately induces senescence. Pioneering work has contributed to our understanding of oxidative stress in the occurrence and development of IDD [32–34]. As confirmed in a previous study, NOX4, a key gene in cell oxidative stress, was significantly upregulated after the oxygen concentration increased [12]. Obviously, our conclusions were consistent with those of the previous research. Additionally, the MAPK and NF-κB signaling pathways were shown to be involved in NP cell senescence after NOX4 activation [12]. Consequently, the significantly change of EZH2, NOX4 and p16 in NP cells with aging suggested that vacuolated NP cells trapped into senescence and oxidative stress. Once the number of these senescent NP cells increase, the homeostasis balance of IVDs will be disrupted, which further leads to IDD [26].

**Fig. 7:**
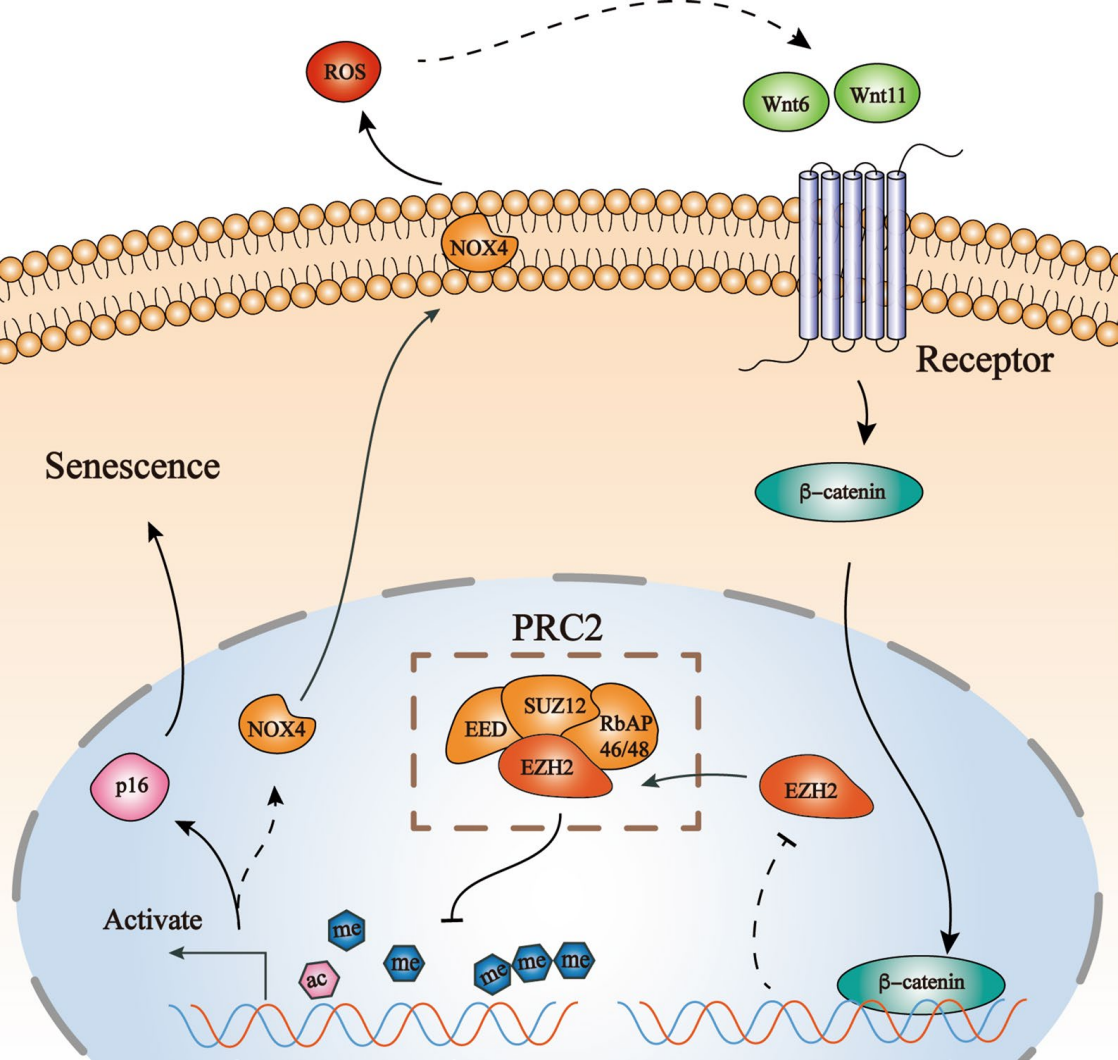
The positive feedback loop of EZH2 and NOX4 was involved in NP cell senescence. Changes in EZH2 regulated activation of the upstream promoter of NOX4 through H3K27me3 and H3K27ac. Overexpression of NOX4 inhibited the expression of EZH2 by activating the canonical Wnt/β-catenin pathway, further inducing NP cell senescence

EZH2 overexpression gives rise to cell immortalization and maintains resistance to senescence by suppressing the transcription of some genes, such as phosphatase and tensin homolog [35]. Depletion of EZH2 activates CDKN1A, thus contributing greatly to cell senescence and apoptosis [36–39]. NOX4 was significantly increased by siEZH2 and decreased by the EZH2 vector in our research. Considering the posttranscriptional modification of histone induced by EZH2, we verified the direct connection between the NOX4 promoter and H3K27me3. H3K27me3 represses gene expression by epigenetic modifications. In addition, H3K27ac plays a role opposite that of H3K27me3 by activating gene transcription [23, 40]. Obviously, our results suggested that NOX4 is one of the genes regulated by H3K27me3 and H3K27ac. In other words, EZH2 regulated NOX4 in NP cells via H3K27me3 and H3K27ac (Fig. 7). In the future, the regulation of the whole genome of NP cells by H3K27me3 will be a potential direction to explore.

The canonical Wnt/β-catenin signaling pathway plays an important role in promoting stem cell proliferation and differentiation [41] and is closely related to not only disc formation and spinal cord development [42] but also joint degeneration and matrix degeneration [43–45]. Studies have shown that the expression levels of the Wnt/β-catenin pathway-related genes axin2, cyclin D1, and c-Myc were significantly increased in degenerative NP cells, and morphology changes in NP cells were induced by the Wnt/β-catenin pathway at the early stages in vitro [46]. Furthermore, EZH2 is directly and physically related to β-catenin [47, 48], which is supported by gene mapping research [49]. In addition, the overexpression of EZH2 inhibited the Wnt pathway by inhibiting Wnt1, Wnt6, Wnt10a and Wnt10b, thereby inhibiting the osteogenic differentiation of MSCs [50]. Conversely, the inhibition of EZH2 further led to Wnt pathway blockade, as described by Chen et al. [51]. However, all of these studies propose that EZH2 regulates the Wnt pathway upstream. Other studies have shown that the expression of the Wnt/Myc pathway is inhibited after DNA damage, further reducing the transcription of EZH2 [16]. In our study, significant upregulation of β-catenin expression after NOX4 overexpression confirmed that the classic Wnt/β-catenin pathway was activated because of the excessive amount of NOX4. The results of our experiment indicate that the Wnt pathway could also regulate the changes in EZH2 upstream. As shown in the PCR array results, significantly high expression of the Wnt pathway-related ligands Wnt11 and Wnt6, as well as the significant downregulation of the antagonist Wif1, also indirectly confirmed this hypothesis. High Wnt11 expression inhibits the proliferation of adipose-derived stem cells through cell cycle arrest, and they can then differentiate into the NP cell phenotype [52]. Therefore, we speculated that the significant increase in Wnt11 induced by NOX4 overexpression was an important factor for the induction of NP cell senescence. The specific mechanism of Wnt11 involvement in the aging of NP cells and disc degeneration is still unclear, and whether nonclassical Wnt pathways are involved in this process requires further research and exploration. Wnt6, a member of the Wnt protein family, is always coexpressed with Wnt10a. These molecules are both highly expressed in carcinogenesis and cell proliferation [53]. Nevertheless, how Wnt6 is activated and further inhibits EZH2 after NOX4 overexpression remains unclear. The foregoing discussion illustrates that downregulation of the EZH2 switch is a vicious cycle (Fig. 7), which further promotes NOX4-related oxidative stress in NP cells and then accelerates their senescence, finally inducing IDD.

There are several limitations in our study. The conditions used in vitro are quite different from those used in vivo, especially the oxygen concentration, nutrition, osmotic pressure and mechanical stress, which introduces variability into our research. On the other hand, the biomechanical properties of the caudal discs are different from those of the lumbar discs, and the biological functions of the NP cells may be different.

## Conclusions

In summary, both EZH2 and NOX4 are involved in natural disc degeneration. Firstly, we found that changes in EZH2 regulate the activation of the upstream promoter of NOX4 through H3K27me3 and H3K27ac. Subsequently, overexpression of NOX4 inhibited the expression of EZH2 by activating the canonical Wnt/β-catenin signaling pathway, further inducing the aging of NP cells. EZH2 and NOX4 are jointly involved in the regulation of aging of NP cells which provide a new idea for further research on the mechanism of disc degeneration, potential targets for early clinical diagnosis and treatment of IDD.

## Methods

### Age-related IDD in rats

To investigate IDD in aging rats, MRI scanning of coccygeal discs from 2-month-old (2 M) and 10-month-old (10 M) rats was performed. Additionally, cross sections of these discs were obtained, and H&E staining was performed.

### Cell isolation and culture

NP cells were isolated from 2 M rats as previously described [54]. Isolated NP cells were cultured at 37 °C and 5% O_2_ and incubated with DMEM/F12 medium (Thermo Fisher, CA, USA) mixed with 10% fetal bovine serum (FBS) and 1% penicillin–streptomycin (Solarbio, Beijing, CN). After 14 days of culture, the NP cells were digested and reseeded in 25-cm^2^ culture flasks (Corning, NY, USA) for further experiments.

### Aging model induced by H_2_O_2_ and TNF-α

For cell senescence induction, referenced H2O2 and TNF-α treatment protocols were used for our experiment [55, 56]. Briefly, 2 × 10^4^ cells were seed in six-well cell culture plates and cultured in complete medium for 1 day. After adherence, cells were treated with H_2_O_2_ (100 μM, 2 h) or TNF-α (30 ng/ml, 72 h), and then washed with PBS to remove H_2_O_2_ and TNF-α. After these treatments, cells cultured in growth medium for an additional 3 days for subsequent experiments. The control group was free from intervention.

### Reagents

Antibodies against EZH2 (#5246), SAPK1/JNK (#9252), phospho-EZH2 (p-EZH2, #27888), tri-methyl-histone H3 (Lys27) (H3K27me3, #9733), acetyl-histone H3 (Lys27) (H3K27ac, #4353), histone H3 (H3, #4499) and β-catenin (#8480) and a Senescence β-Galactosidase Staining Kit (#9860) were purchased from Cell Signaling Technology (Danvers, MA, USA). Antibodies against NOX4 (ab133303) and p16 (ab189034) were obtained from Abcam (Cambridge, MA, USA). An antibody against β-actin (sc69879) was provided by Santa Cruz Biotechnology (Santa Cruz, CA, USA). Antibodies against Wnt 11 (SAB2700897), Wnt 6 (SAB2108257), and Wif1 (SAB4301685) were obtained from Sigma-Aldrich (Merck KGaA, Darmstadt, DE). Alexa Fluor 488-labeled goat anti-rabbit IgG (H + L), 647-labeled goat anti-rabbit IgG (H + L) (A0423 and A0468), a Reactive Oxygen Species Assay Kit and an EdU Cell Proliferation Kit with Alexa Fluor 647 (C0071S) were purchased from Beyotime (Shanghai, CN). Horseradish peroxidase (HRP)-conjugated secondary antibodies (ZB2305 and ZB2301) were purchased from ZSGB-BIO (Beijing, China). An EZH2 inhibitor (GSK126), NOX4 inhibitor (GKT137831) and Wnt signaling pathway inhibitor (KYA1797K) were both purchased from Selleck Chemicals (Houston, TX, USA). TNF-α (#5178) were also purchased from Cell Signaling Technology (Danvers, MA, USA).

### Micro-MRI

Different aged rats secured in a micro-MRI cradle were anaesthetized with isoflurane and advanced into the magnet (7.0 Tesla, Bruker Biospec 70/20 USR, Bruker BioSpin Corporation Billerica, MA) with the following scanning parameters: sequence: TURBO RARE; weighting: T2; TE: 35 ms; TR: 3000 ms; flip angle: 90; and slice thickness: 0.70 mm. The T2 signal intensity was tested by Sante DICOM Viewer Free (version 5.3).

### EdU incorporation assay

For EdU incorporation, NP cells seeded on coverslips were incubated with DMEM/F12 medium containing EdU (10 μM, Beyotime, Shanghai, CN) for 24 h. After fixation with 4% paraformaldehyde, the cells on the coverslips were incubated in the dark with Click Additive Solution at room temperature for 30 min. Finally, NP cells were incubated in the dark with 1 ml of Hoechst solution (1:1000 dilution) at room temperature for 10 min. Six random fields per well were imaged under a fluorescence microscope (400× magnification, Olympus). The mean percentage of EdU-positive cells was calculated.

### SA-β-gal staining

SA-β-gal staining was performed as previously described [12]. Briefly, NP cells were incubated in 6-well plates (35-mm wells). After fixation, β-galactosidase staining solution was added to each well. The plates were sealed with parafilm and incubated at 37 °C overnight in a dry incubator. Six random fields per plate well were imaged using a phase-contrast microscope (200× magnification, Olympus). Then, the mean percentage of SA-β-gal-positive cells was calculated.

### Lentiviral transfection

NP cells were transfected with small interfering RNA against EZH2 (siEZH2) (Gene Chemistry, Shanghai, CN), scrambled siRNA control, NOX4 vector or EZH2 vector using Lipofectamine 2000 transfection reagent (Invitrogen, Carlsbad, CA, USA). The siEZH2 sequence was 5′-CACAGCAGAAGAACTGAAAGA-3′, and the transfection efficiency was detected by immunofluorescence. A percentage of green fluorescent protein (GFP)-positive cells over 80% was considered successful, and cells transfected with siEZH2 are shown in Additional file 5: Figure S4. The knockdown and overexpression efficiencies were determined by RT-qPCR and immunoblot analysis after 72 h of transfection.

### Immunoblot analysis

Total NP tissue was homogenized in RIPA buffer using a bead homogenizer (Bertin, FR). Cultured cells were washed with PBS (pH = 7.4) before lysis with RIPA buffer and then quantified using a BCA kit (Beyotime). Total protein samples were mixed with loading buffer (Invitrogen) and boiled for 10 min. Equal amounts of protein (30 μg) were separated on 8% or 12% SDS-PAGE gels and transferred to PVDF membranes (Millipore). Primary antibodies against EZH2 (1:1000 dilution), p-EZH2 (1:1000 dilution), NOX4 (1:2000 dilution), H3 (1:1000 dilution), H3K27me3 (1:1000 dilution), H3K27ac (1:1000 dilution), β-actin (1:200 dilution), β-catenin (1:1000 dilution), p16 (1:1000 dilution), Wnt11 (1:400 dilution), Wnt6 (1:500 dilution), Wif1 (1:500 dilution), and SAPK1/JNK (1:1000 dilution) were used. Anti-rabbit IgG-HRP (ZB2305, 1:4000) and anti-mouse IgG-HRP (ZB2301, 1:4000) secondary antibodies were then applied. The protein bands were detected by electrochemiluminescence regents (Thermo Scientific). The optical density (OD) of the protein bands was measured by ImageJ software (National Institutes of Health, USA). The protein expression level of β-actin was used for normalization.

### Immunohistochemistry analysis

Deparaffinized IVD sections were incubated with antibodies against NOX4 (1:500 dilution), EZH2 (1:250 dilution), and p16 (1:500 dilution) at 4 °C overnight. Then, the sections were incubated with HRP-conjugated secondary antibodies at room temperature for 1 h. Cell nuclei were counterstained with hematoxylin. Images of these sections were obtained with a microscope (Olympus). Six random fields per section well were imaged. The integrated optical density (IOD) was measured by Image-Pro Plus 6.0. The mean OD (IOD/area) was used to evaluate the expression of the target proteins.

### Immunofluorescence assay

NP cells seeded in cell culture dishes (15 mm) were fixed with 4% paraformaldehyde for 15 min. For frozen sections, the cells were fixed directly with paraformaldehyde. After permeabilization and antigen blocking, the cells and frozen sections were incubated with primary antibodies against p16 (1:200 dilution), EZH2 (1:50 dilution), and NOX4 (1:250 dilution) overnight at 4 °C. After rinsing, the cells and frozen sections were incubated with Alexa Fluor 647 and 488 dye-conjugated secondary antibodies (1:1000 dilution) in the dark for 1 h. Cells without coincubation with the primary antibodies served as a negative control. Pictures were taken under a confocal microscope (Lecia, Wentzler, Germany).

### Real-time quantitative PCR (RT-qPCR)

Total RNA was isolated from cultured NP cells and reverse transcribed into cDNA as previously described [12]. RT-qPCR was carried out in triplicate using a ViiATM7 Real-Time PCR system (Thermo Scientific), and a SYBR^®^ Premix Ex Taq™ II kit (Takara Bio) was applied for the reactions. The relative mRNA expression levels were quantified using the comparative C_T_ method. GAPDH mRNA expression was used for normalization. The primers used in this research are presented in Table 1.

**Table 1:** Primer sequences used in the real-time quantitative PCR analysis

Target gene	Forward primer	Reverse primer
EZH2	GAATGGAAGCAGCGAAGGATACAG	CAAGTCACTGGTCACTGAACACTCC
p16	CGTCGTGCGGTATTTGCGGTATC	CGTTGCCAGAAGTGAAGCCAAGG
NOX4	ATGGTGGTGGTATTGTTCCTCAT	CAGCAGCAGCAGCATGTAGA
GAPDH	CGGCAAGTTCAACGGCACAGT	CGACATACTCAGCACCAGCATCAC
β-Actin	TCAGGTCATCACTATCGGCAAT	AAAGAAAGGGTGTAAAACGCA
β-Catenin	CATTACAACTCTCCACAACC	CAGATAGCACCTTCAGCA
Wnt11	ATGGCATCAAGTGGCTGGCATT	ATGGCATCAAGTGGCTGGCATT
Wnt6	ATGCTGCTGCTGCTGCTCTTGC	AACGGAACTGGAACTGACATTCTCG
Mapk8	ACAGTGAGCAGAGCAGGCATAGT	TTGTCAGGAGCAGCACCATTCTTAC
Wif1	CCAGGCGAGAACTTCACAAGCA	TAGCAGGAGCAGGCAAGGTAGG

*NOX4* NADPH oxidase 4, *EZH2* enhancer of zeste homolog 2, *Wnt6* Wnt family member 6, *Wnt11* Wnt family member 11, *Wif1* WNT inhibitory factor 1, *Mapk8* mitogen-activated protein kinase 8

### PCR array

An RT^2^ Profiler PCR Array [96-well format and 384-well (4 × 96) format, Cat. no. 330231, Qiagen] was applied for our analysis. A total of 10 µl of RT^2^ SYBR Green Mastermix, nuclease-free H_2_O and cDNA was added to each well. The cycling program included preincubation for 10 min at 95 °C (1 cycle), denaturation for 15 s at 95 °C, and annealing and extension for 1 min at 60 °C (40 cycles). Genes that changed significantly (p < 0.05) are presented in Fig. 6b. The original data are presented in Additional file 4: Table S1.

### ROS measurement

ROS levels in NP cells were analyzed by using Reactive Oxygen Species Assay Kit (Beyotime, Shanghai, CN). NP cells transfected with lentiviral in six-well cell culture plates were treated with DCFH-DA (10 μM) at 37 °C for 20 min. Cells treated with Rosup (50 μg/ml, 20 min) was served as a positive control. The excitation and emission wavelength were measured at 488 nm and 525 nm. Samples were analyzed using a Gallios Flow Cytometer (Beckman Coulter, Brea, CA, USA) and at least 5000 cells were collected per sample. Data were analyzed using the Kaluza For Gallios 1.0 software and FlowJo 10.0 software.

### Cell cycle analysis

Flow cytometry (FCM) were applied to test cell cycle arrest induced by H_2_O_2_ and TNF-α. After trypsinization, detached cells were fixed in 70% ethanol at 4 °C overnight. After washing with PBS, cells were separately stained with 50 μg/ml RNase A and 50 μg/ml PI at 37 °C for 30 min. Samples were analyzed using a Gallios Flow Cytometer (Beckman Coulter, Brea, CA, USA) and at least 8000 cells were collected per sample. Data were analyzed using the Kaluza For Gallios 1.0 software.

### Chromatin immunoprecipitation assay (ChIP)

A ChIP assay was performed with reagents, antibodies, and protocols from Cell Signaling Technology. Rabbit polyclonal antibodies against H3K27me3 (#9733, 1:50 dilution) and H3K27ac (#4353, 1:25 dilution) were applied for the assay. PCR was performed with three primer sets [57] spanning the region of the NOX4 proximal promotor (≈ 800 bp). ‘Input’ (DNA purified by sonication), rabbit IgG antibody, and negative controls (no antibody) were used. Melting curves and gel electrophoresis were applied to analyze the specificity of the PCR product.

### Statistical analysis

All data were obtained from at least three independent measurements. The data are shown as the mean with SEM. A two-tailed Student’s t test was used for comparisons between two independent samples. One-way ANOVA followed by Tukey’s post hoc test was used for data analysis among three or more groups. The data were analyzed by GraphPad Prism 8, and p < 0.05 was considered significant.

## Supplementary information

**Additional file 1: Figure S1.** Mean fluorescence intensity of NOX4 and EZH2 in NP frozen sections in 2 M and 10 M rats. n = 3. ^**^p < 0.01.

**Additional file 2: Figure S2.** Immunohistochemistry staining of p16 in NP paraffin sections in 2 M and 10 M rats. Magnification, 200× and 400×.

**Additional file 3: Figure S3.** SA-β-Gal and EdU staining of cells transfected with siCtrl and siEZH2 under a phase-contrast microscope and a fluorescence microscope.

**Additional file 4: Table S1.** Original data of PCR array analysis.

**Additional file 5: Figure S4.** Cells transfected with siEZH2 were observed under a phase-contrast microscope (a) and a fluorescence microscope (b). The rate of positive cells ≈ 86%.

## Data Availability

All data generated or analyzed during this study are included in the article.

## Abbreviations

ECM: extracellular matrix
EZH2: enhancer of zeste homolog 2
H3K27ac: histone H3 lysine 27 acetylation
H3K27me3: histone H3 lysine 27 trimethylation
IDD: intervertebral disc degeneration
IVDs: intervertebral discs
NOX4: NADPH oxidase 4
Mapk8: mitogen-activated protein kinase 8
NP: nucleus pulposus
p-EZH2: phosphorylation of EZH2
PRC2: polycomb repressive complex 2
SASP: senescence-associated secretory phenotype
Wnt6: Wnt family member 6
Wnt11: Wnt family member 11
Wif1: WNT inhibitory factor 1
