# Supplementary figures and images for: A positive feedback loop between EZH2 and NOX4 regulates nucleus pulposus cell senescence in age-related intervertebral disc degeneration
Source: Cell Div. 2020 Feb 1;15:2. doi: 10.1186/s13008-020-0060-x (PMC6995653; doi:10.1186/s13008-020-0060-x)

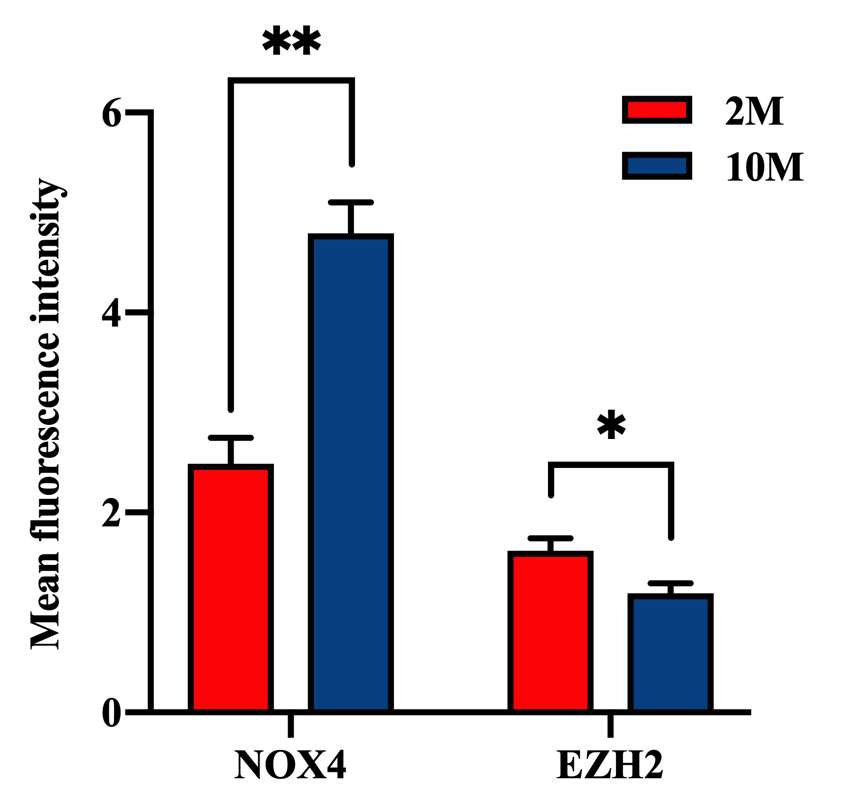

Supplement: Supplementary file 1 — Additional file 1: Figure S1. Mean fluorescence intensity of NOX4 and EZH2 in NP frozen sections in 2 M and 10 M rats. n = 3. **p < 0.01. [file 13008_2020_60_MOESM1_ESM.tiff]

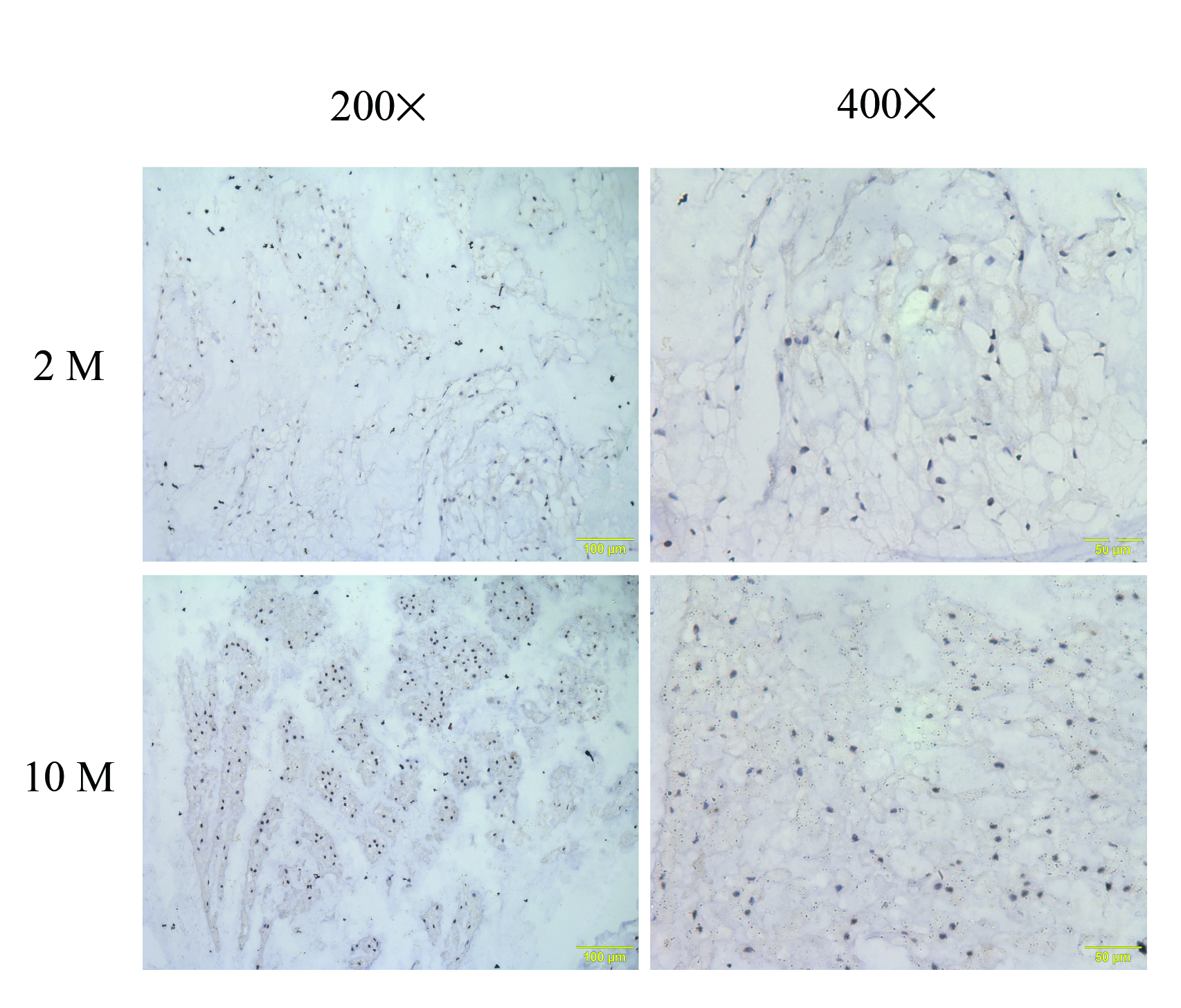

Supplement: Supplementary file 2 — Additional file 2: Figure S2. Immunohistochemistry staining of p16 in NP paraffin sections in 2 M and 10 M rats. Magnification, 200× and 400×. [file 13008_2020_60_MOESM2_ESM.tif]

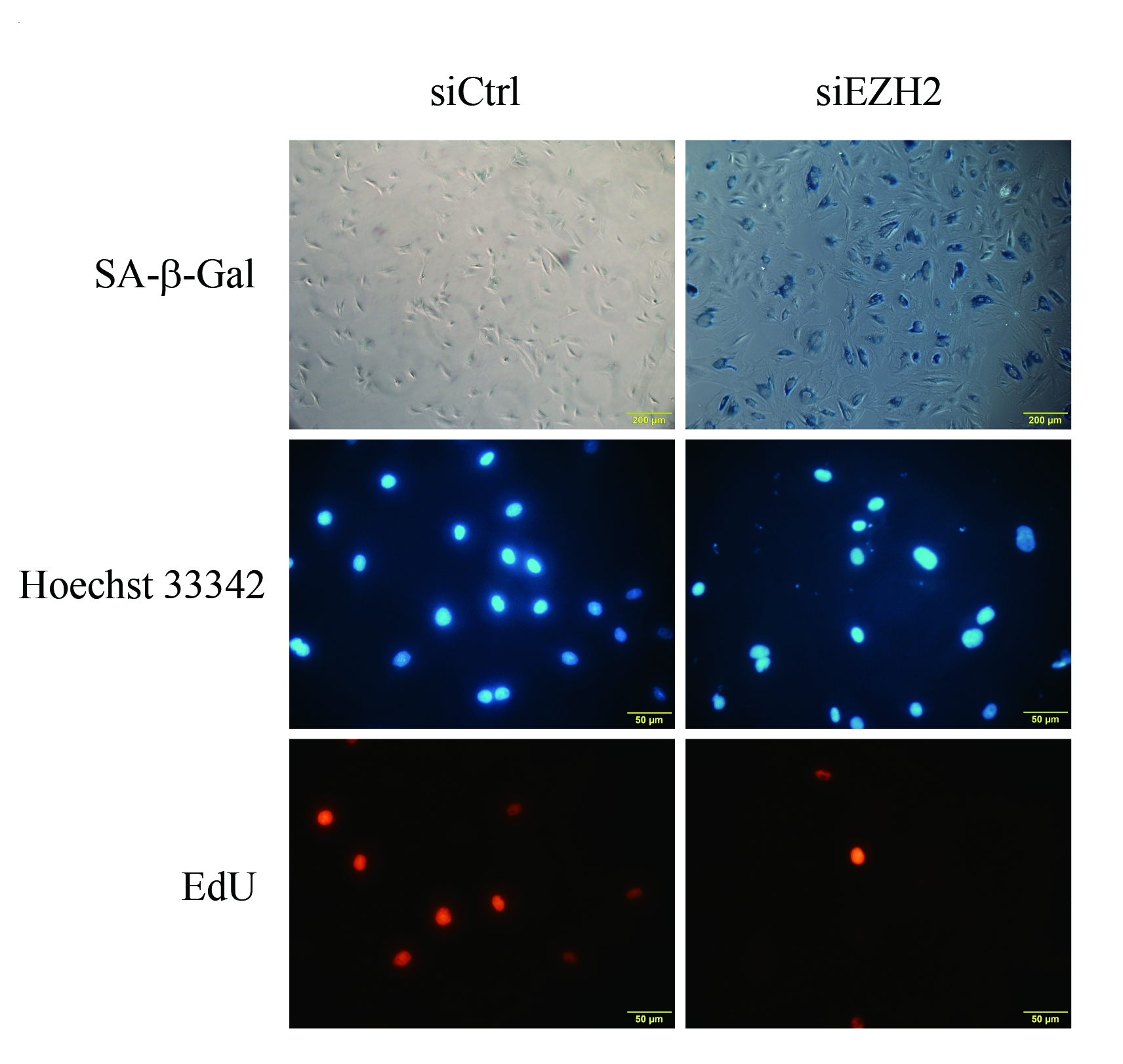

Supplement: Supplementary file 3 — Additional file 3: Figure S3. SA-β-Gal and EdU staining of cells transfected with siCtrl and siEZH2 under a phase-contrast microscope and a fluorescence microscope. [file 13008_2020_60_MOESM3_ESM.tif]

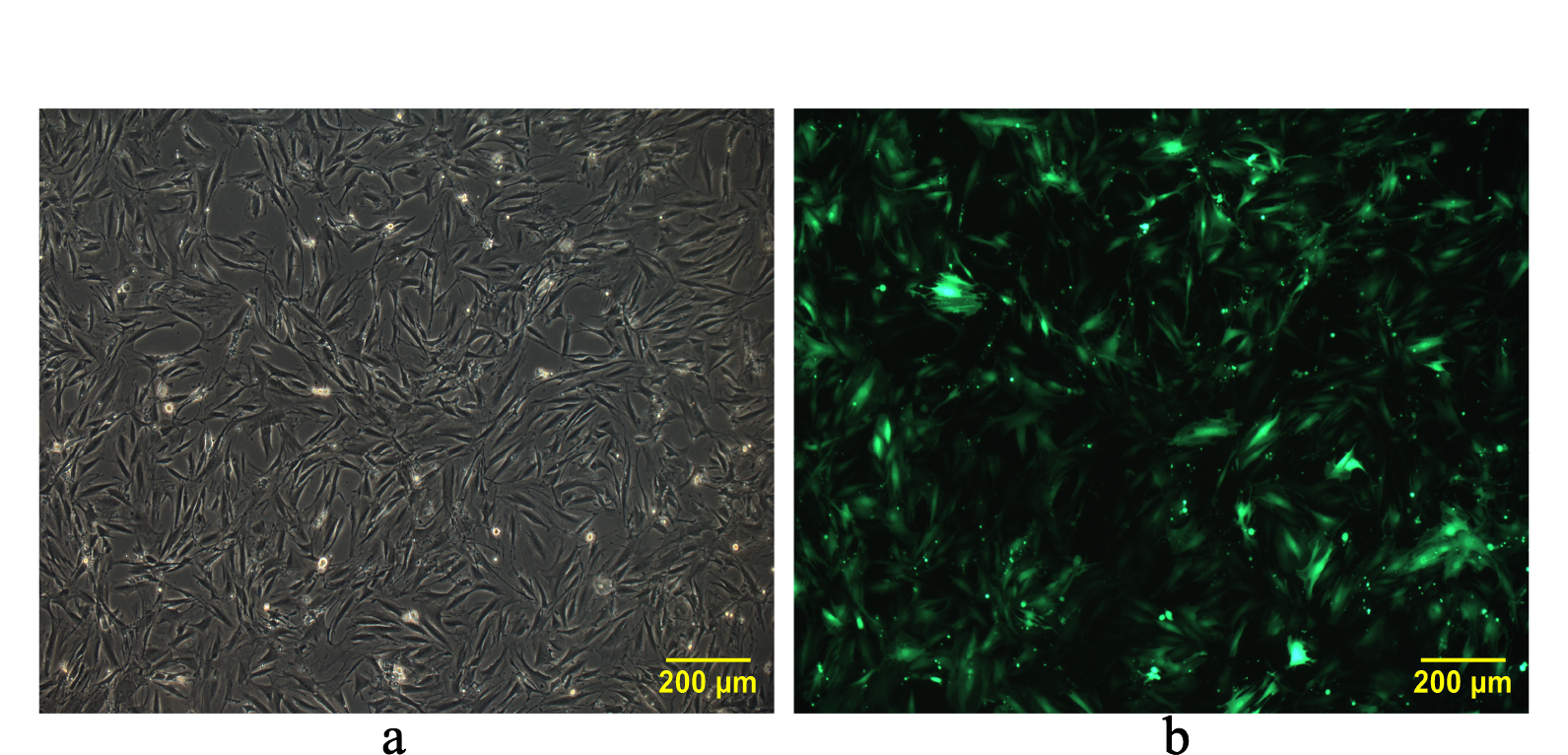

Supplement: Supplementary file 5 — Additional file 5: Figure S4. Cells transfected with siEZH2 were observed under a phase-contrast microscope (a) and a fluorescence microscope (b). The rate of positive cells ≈ 86%. [file 13008_2020_60_MOESM5_ESM.tif]
